# Acute lower respiratory tract infections: Symptoms, findings and management in Danish general practice

**DOI:** 10.1080/13814788.2019.1674279

**Published:** 2019-10-25

**Authors:** Line Sloth Hansen, Jesper Lykkegaard, Janus Laust Thomsen, Malene Plejdrup Hansen

**Affiliations:** a Center for General Practice, Aalborg University, Aalborg, Denmark;; b Audit Project Odense, Research Unit of General Practice, Institute of Public Health, University of Southern Denmark, Odense, Denmark

**Keywords:** Acute bronchitis, C-reactive protein, general practice, lower respiratory tract infections, pneumonia

## Abstract

**Background:** Acute lower respiratory tract infections (LRTIs) are among the most common infections managed in general practice.

**Objectives:** To describe differences in reported symptoms, findings and management of patients diagnosed with acute LRTIs, and to explore possible associations between these findings and being diagnosed with pneumonia.

**Methods:** During one winter season (2017 or 2018), a prospective registration of patients diagnosed with either acute bronchitis (ICPC-2: R78) or pneumonia (ICPC-2: R81) was conducted in Danish general practice for 20 days. A 42 item registration chart was filled in for each patient. Descriptive statistics, Pearson's chi-square test and multiple logistic regressions were used for data analysis.

**Results:** In total, 70 general practices participated with 1384 patients registered. Patients diagnosed with pneumonia were more often reported as having a fever, dyspnoea, increased purulent sputum, abnormal pulmonary auscultation/chest retractions, and were more often assessed as unwell by the healthcare professional, than those diagnosed with acute bronchitis. Very few patients had a chest X-ray. Contrary, most patients had a C-reactive protein (CRP) test performed (pneumonia: 83%; acute bronchitis: 71%). Respectively, 93% and 20% of patients were treated with antibiotics. Having a fever, an abnormal pulmonary auscultation/chest retractions or being assessed as unwell increased the likelihood of the diagnosis pneumonia at least fivefold. Even a slightly elevated CRP (≥11 mg/L) was positively associated with being diagnosed with pneumonia.

**Conclusion:** Danish healthcare professionals are highly influenced by symptoms, signs and CRP tests when diagnosing patients with acute LRTIs in general practice.

KEY MESSAGES

- Having a fever, abnormal auscultation/retractions or being assessed as unwell by the healthcare professional increased the likelihood of being diagnosed with pneumonia at least fivefold.
- Few patients had a chest X-ray before being diagnosed with pneumonia.
- Even a slightly elevated CRP (≥11 mg/L) was associated with being diagnosed with pneumonia.

## Introduction

Antimicrobial resistance is one of the greatest threats to global public health and the World Health Organisation warns against a return to a pre-antibiotic era [1]. Higher prevalence of resistance among human pathogens increases the risk of uncontainable infections, prolonged illness and hospital stay, increased mortality, and consequently increased health care costs [2]. Antibiotic use is the main driver of antibiotic resistance, why addressing the excessive and inappropriate use of antibiotics is essential [3]. In Denmark, general practice accounts for about 75% of the total human antibiotic consumption [4]. Acute lower respiratory tract infections (LRTIs) are among the most common infections managed in Danish general practice [5], with pneumonia being a common indication for antibiotic prescriptions [6].

According to Danish and international recommendations, patients with suspected pneumonia should, in general, be treated with antibiotics [5]. Contrary, acute bronchitis is most often considered a viral infection and thus most patients will not benefit from antibiotic treatment [5]. However, it can be difficult to differentiate pneumonia from other LRTIs by means of symptoms and signs [7], and the point-of-care test (POCT) named C-reactive protein (CRP) has been used since 1999 in Danish general practice [8]. Evidence exists that CRP-testing can reduce antibiotic prescribing for acute respiratory tract infections [9] and many guidelines recommend CRP-testing in patients presenting with symptoms of an acute LRTI [5,10]. However, as CRP is a non-specific marker of inflammation, it is challenging to set a specific cut-off value for treatment with antibiotics. Also, imaging can be used as a supportive diagnostic tool for diagnosing pneumonia, with chest X-ray being the most commonly used. However, diagnostic imaging is far from always used in patients suspected for pneumonia due to low availability, high radiation dose, and high costs. In summary, a great deal of diagnostic uncertainty exists when dealing with patients with acute LRTIs in general practice and this may lead to too many people being diagnosed with pneumonia and thus resulting in inappropriate use of antibiotics [11].

This study aimed to describe differences in reported symptoms, findings and management of patients diagnosed with either acute bronchitis or pneumonia in general practice, and to explore possible associations between the symptoms, findings, and CRP level and being diagnosed with pneumonia.

## Methods

### Setting

This prospective, cross-sectional study is part of a larger quality improvement project with the overall aim of improving diagnosis and treatment of acute respiratory tract infections in Danish general practice. Both GPs and practice nurses were asked to participate in the project, as many patients with acute minor illnesses, such as acute respiratory tract infections, are taken care of by a practice nurse in Danish general practice. The participating general practices originated from three Danish Regions. During winter 2017, general practices in the North Denmark Region and the Region of Southern Denmark registered all patients presenting with symptoms of an acute respiratory tract infection for 20 days. In winter 2018, general practices in the Central Denmark Region performed the registrations. Only patients who consulted the practice for the first time for the current infection were included. Home visits and telephone consultations were not included. Registration was performed according to the Audit Project Odense (APO) method, using a registration chart with 42 items [12] (Supplementary Material). All symptoms and findings were simply listed in the registration chart, with no specific definitions provided. However, all participating healthcare professionals, i.e. general practitioners and practice nurses, were provided with a guide instructing them on how to fill in the registration chart and specifying that the diagnoses given should be based on the International Classification of Primary Care (ICPC-2). It was recommended to perform the registration during or immediately after each consultation.

### Ethics

All general practitioners and practice nurses consented to the study. Only anonymised patient data were obtained and ethics approval was not indicated according to Danish law. The project is registered at the University of Southern Denmark, Denmark (ID SDU 10.169).

### Subjects

In total, 8232 patients with acute respiratory tract infection were registered. However, only patients diagnosed with either acute bronchitis (ICPC-2 code R81) or pneumonia (ICPC-2 code R78) comprise the study population for the present study. No formal diagnostic criteria had to be met and the diagnosis given was solely based on the clinical judgement of the participating healthcare professional. Patients diagnosed with exacerbation of chronic obstructive pulmonary disease (ICPC-2 codes R95, R79) was not included in the analysis (*n* = 197).

### Data

The general practitioners/practice nurses were asked to tick off if any of the following symptoms and findings were registered: fever (>38.5 °C), cough, dyspnoea, increased purulent sputum, abnormal pulmonary auscultation/chest retractions, and if the healthcare professional deemed the patient unwell or as a weakened/multimorbid patient. Also, it was registered if a CRP test (including the value in mg/L) and/or a chest X-ray was performed, and if any antibiotic treatment was provided (Supplementary Material).

In addition, a short questionnaire focussing on practice characteristics and personal information was completed by each of the participating general practitioners and practice nurses.

### Statistical analysis

Categorical variables were presented as numbers and percentages, and metric variables were presented as medians and percentiles. Pearson’s chi-square test was applied with a 5% significant level to test for independence. Multiple logistic regressions were performed to analyse the association between the symptoms, findings, and CRP values and being diagnosed with pneumonia. The odds ratios (OR) of being diagnosed with pneumonia were adjusted for possible confounders (gender, age and weakened/multimorbid patient). As effect modification was suspected, interactions between the various symptoms were tested. In the descriptive statistics missing values are reported in the respective tables and pairwise deletion was used in the logistic regressions.

All statistical analyses were conducted using SPSS Statistics 25 [13].

## Results

### Baseline characteristics of subjects

In total, 70 general practices agreed to participate. Table 1 demonstrates the characteristics of the 158 general practitioners and 56 practice nurses managing patients diagnosed with either acute bronchitis or pneumonia. Compared to the total population of Danish GPs, the participating GPs were more likely to be female, to be younger, and to work in partnership practices [14]. Participating nurses were older than the national average age for nurses [15].

**Table 1.** Characteristics of the 158 participating general practitioners and 56 practice nurses managing patients with the diagnosis of either pneumonia or acute bronchitis (2017/2018).

	General practitioners (*n* = 158)	Practice nurses (*n* = 56)
Gender (female)^a^	103 (65.2)	53 (94.6)
*Missing*	–	3 (5.4)
Age (years)^b^	46 (39–57)	49 (41–58)
Region^a^		
North Denmark Region	68 (43.0)	25 (44.6)
Central Denmark Region	36 (22.8)	14 (25.0)
Region of Southern Denmark	54 (34.2)	17 (30.4)
Practice type^a^		
Solo practice	23 (14.6)	15 (26.8)
Partnership practice	120 (75.9)	32 (57.1)
Collaboration practice	12 (7.6)	8 (14.3)
*Missing*	3 (1.9)	1 (1.8)

^a^Values presented as *n* (%).

^b^Values presented as median (25th and 75th percentile).

A total of 1384 patients were diagnosed with an acute LRTI, of which 50.5% were diagnosed with acute bronchitis and 49.5% with pneumonia (Table 2). Most patients were adults, and slightly more female patients were registered. More children (≤5 years) were diagnosed with acute bronchitis than pneumonia. Contrary, elderly patients (>65 years) were more commonly diagnosed with pneumonia than acute bronchitis.

**Table 2.** Baseline characteristics of the 1384 patients diagnosed with either pneumonia or acute bronchitis (2017/2018).

	Patients diagnosed with pneumonia (*n* = 685)	Patients diagnosed with acute bronchitis (*n* = 699)
Gender (female)^a^	365 (53.3)	405 (57.9)
*Missing*	1 (0.1)	–
Age (years)^a^		
≤ 5 years	83 (12.1)	162 (23.2)
6–30 years	83 (12.1)	99 (14.2)
31–65 years	248 (36.2)	285 (40.8)
>65 years	269 (39.3)	149 (21.3)
*Missing*	2 (0.3)	4 (0.6)
Weakened or multimorbid^a^	84 (12.3)	26 (3.7)
Duration of symptoms before contact (days)^b^	6 (3–10)	7 (4–14)

^a^Values presented as *n* (%).

^b^Values presented as median (25th and 75th percentile).

### Management of patients with acute bronchitis or pneumonia

The most frequently reported symptoms among patients diagnosed with acute bronchitis or pneumonia were cough, fever, dyspnoea, and increased purulent sputum (Table 3). Patients diagnosed with pneumonia were more often reported with a fever, dyspnoea, and increased purulent sputum, respectively, than those diagnosed with acute bronchitis. Also, patients diagnosed with pneumonia were more often reported with abnormal pulmonary auscultation/chest retractions and were more often assessed as unwell by the healthcare professional than those diagnosed with acute bronchitis.

**Table 3.** Symptoms, findings, diagnostic tests and antibiotic treatments given to 1384 patients diagnosed with either pneumonia or acute bronchitis (2017/2018).

	Patients diagnosed with pneumonia (*n* = 685)	Patients diagnosed with acute bronchitis (*n* = 699)	Chi-square value, *p* value
Reported symptoms^a^			
Cough	643 (93.9)	682 (97.6)	χ2 = 10.8,*p* = 0.001
Fever (tp.>38.5 °C)	435 (63.5)	239 (34.2)	χ2 = 115.4, *p* < 0.001
Dyspnoea	265 (38.7)	169 (24.2)	χ2 = 33.4, *p* < 0.001
Increased purulent sputum	224 (32.7)	148 (21.2)	χ2 = 23.1, *p* < 0.001
Findings^a^			
Abnormal auscultation/chest retractions	460 (67.2)	251 (35.9)	χ2 = 130.6, *p* < 0.001
Generally unwell	309 (45.1)	110 (15.7)	χ2 = 131.6, *p* < 0.001
CRP test performed^a^^–c^	569 (83.1)	499 (71.4)	
CRP ≤10 mg/L	34 (6.0)	262 (52.5)	χ2 = 160.8, *p* < 0.001
CRP 11–25 mg/L	56 (9.8)	132 (26.5)	χ2 = 32.1, *p* < 0.001
CRP 26–49	112 (19.7)	54 (10.8)	χ2 = 23.4, *p* < 0.001
CRP ≥50 mg/L	360 (63.3)	48 (9.6)	χ2 = 260.1, *p* < 0.001
*Missing CRP value*^d^	7 (1.2)	3 (0.6)	
Chest X-ray^a^	40 (5.8)	22 (3.1)	
Antibiotic prescribed^a^^,b^	635 (92.7)	136 (19.5)	
Phenoxymethylpenicillin	412 (64.9)	74 (54.4)	
Amoxicillin ± clavulanic acid	93 (14.6)	29 (21.3)	
Macrolide	104 (16.4)	28 (20.6)	
Other antibiotics	26 (4.1)	5 (3.7)	
*Missing*^e^	2 (0.3)	7 (1.0)	

^a^Values presented as *n* (%). The percentage is based on the total number of patients diagnosed with either pneumonitis or acute bronchitis.

^b^In the subgroups the percentage is based on the number of patients with the CRP test performed or antibiotic prescribed.

^c^CRP values are stratified in four groups.

^d^CRP test performed but CRP-value is missing.

^e^Information about antibiotic prescription is missing.

Very few patients had a chest X-ray. However, most patients had a CRP test performed (pneumonia: 83.1%; acute bronchitis: 71.4%). Overall a difference in the distribution of CRP level groups (≤10, 11–25, 26–49, ≥50 mg/L) between those diagnosed with acute bronchitis and pneumonia was observed (χ^2^=335.1, *p* < 0.001) (Data not shown). Most patients diagnosed with pneumonia had a CRP above 26 mg/L, while most patients diagnosed with acute bronchitis had a CRP below 25 mg/L. Differences between CRP level groups (≤10, 11–25, 26–49, ≥50 mg/L) for those diagnosed with acute bronchitis and pneumonia, respectively, are described in Table 3.

Antibiotics were prescribed for almost all patients diagnosed with pneumonia (93%) and about one fifth of patients diagnosed with acute bronchitis. Phenoxymethylpenicillin was the most commonly prescribed antibiotic, which is in accordance with Danish guidelines.

### Patients diagnosed with pneumonia

The symptoms fever, dyspnoea, and increased purulent sputum, and the findings of abnormal pulmonary auscultation/chest retractions and being assessed as unwell were all positively associated with being diagnosed with pneumonia compared to acute bronchitis (Table 4). When patients reported/presented with fever they were almost five times more likely to be diagnosed with pneumonia (odds ratio (OR) = 4.6; 95% confidence interval (CI) 3.6–5.9). Fever was found to cause-effect modification increasing the association between reporting the symptoms dyspnoea and increased purulent sputum and being diagnosed with pneumonia (data not shown). Also, the higher number of symptoms, the more likely patients were diagnosed with pneumonia (two symptoms: OR = 2.5; 95% CI 1.9–3.3; three symptoms: OR = 4.7; 95% CI 3.4–6.6; four symptoms: OR = 13.6; 95% CI 6.6–27.7) (data not shown).

**Table 4.** Associations between reported symptoms, findings and CRP values and being diagnosed with pneumonia.

	Crude OR (95% CI)	Adjusted OR^a^ (95% CI)
Symptoms^b^		
Cough	0.4 (0.2–0.7)	0.3 (0.2–0.7)
Fever (tp.>38.5 °C)	3.3 (2.7–4.2)	4.6 (3.6–5.9)
Dyspnoea	2.0 (1.5–2.5)	1.8 (1.4–2.4)
Increased purulent sputum	1.8 (1.4–2.3)	1.5 (1.1–1.9)
Findings^b^		
Abnormal auscultation/chest retractions	3.6 (2.9–4.6)	5.6 (4.3–7.3)
Generally unwell	4.4 (3.4–5.7)	5.5 (4.1–7.3)
CRP		
CRP ≤10 mg/L	Ref.	Ref.
CRP 11–25 mg/L	3.1 (1.9–5.0)	3.1 (1.9–5.1)
CRP 26–49 mg/L	16.1 (10.0–26.1)	16.9 (10.3–27.7)
CRP ≥ 50 mg/L	57.8 (36.2–92.2)	57.7 (35.7–93.2)

OR: odds ratio of being diagnosed with pneumonia; CI: confidence interval; Ref: reference.

^a^Adjusted for gender, age and patient assessed as weakened/multimorbid.

^b^References were not having the symptom or the finding (yes/no).

The likelihood of being diagnosed with pneumonia increased with increasing CRP level (Table 4).

## Discussion

### Main findings

Patients diagnosed with pneumonia were more often reported as having a fever, dyspnoea, increased purulent sputum, abnormal pulmonary auscultation/chest retractions, and were more often assessed generally unwell by the healthcare professional, than those diagnosed with acute bronchitis. Having a fever, abnormal pulmonary auscultation/chest retractions or being valued as unwell increased the likelihood of being diagnosed with pneumonia at least fivefold. Very few patients presenting with symptoms of an acute LRTI had a chest X-ray performed. Contrary, most patients had a CRP test performed, and even a slightly elevated CRP test (≥11 mg/L) was positively associated with being diagnosed with pneumonia.

### Strengths and limitations

Projects based on the APO-method have been carried out in Danish general practices since 1989 and on very diverse issues like treatment of hypertension, preventive home visits and acute respiratory tract infections [16–18]. However, some limitations have to be kept in mind when interpreting the results of this study.

First, it is voluntary to participate in APO audits and one can argue that the results do not necessarily reflect the average management of patients with LRTIs in Danish general practices. The participants may have been more interested in quality development and in the topic being investigated than health care professionals in general [19], which could have prompted increased awareness of evidence-based management of patients with LRTIs.

Second, a registration chart with predefined variables was used in this study and it is not possible to explore the accuracy of the reported symptoms or the diagnosis given. For example, the variables ‘abnormal pulmonary auscultation/chest retractions’ and weakened/multimorbid patient included two findings, which makes it impossible to know which one of the findings patients actually presented with, or even if they presented with both findings. Also, fever (temperature >38.5 °C) was registered for 63.5% and 34.2% of patients diagnosed with pneumonia and acute bronchitis, respectively. However, it is not possible to identify when an exact temperature was measured and when the presence of fever was solely based on a subjective assessment of either the patient or the health care professional.

Also, the risk of missing valuable information (symptoms and findings not included in the chart) needs to be mentioned. However, a major strength of using these simple registration charts is the opportunity to easily perform the registration during the consultation, which enables GPs and practice nurses to work according to their usual routine [20].

Finally, it is well known that health care professionals often first decide if antibiotic treatment is indicated or not – and then subsequently label the patient with the most suitable diagnosis. As Howie [21] stated back in 1972 ‘There are occasions when the diagnostic label attached to consultation is a rationalisation of the management decision made, rather than the determinant of it.’ Consequently, when interpreting the results from this study one has to keep in mind that we can only report on ‘a picture’ of the management of patients diagnosed with either acute bronchitis or pneumonia in Danish general practices, and not necessarily report on the correctness of the diagnoses given.

### Interpretation of the study results in relation to existing literature

In accordance with other studies, we found a large overlap of symptoms in patients diagnosed with either acute bronchitis or pneumonia [22]. Also, previously conducted research has demonstrated that both fever and dyspnoea are associated with being diagnosed with pneumonia [11,22]. Importantly, we found that reported fever was found to cause-effect modification of the symptoms dyspnoea and increased purulent sputum. Thus, the best way to describe which symptoms precede the diagnosis of pneumonia is probably not to report a single symptom but to report on a combination of these symptoms.

Being assessed as unwell by the attending healthcare professional was positively associated with being diagnosed with pneumonia. The assessment of patients’ general condition is difficult to define and this subjective assessment has not previously been described in the international literature in relation to the management of patients with acute LRTI. Importantly, we report on the healthcare professionals clinical impression of the patient’s condition, as it was left entirely to the participating GPs and nurses to deem if the patient was unwell.

There is good evidence that most patients with acute bronchitis do not benefit from antibiotic treatment [23]. Still, in this Danish study, about one-fifth of patients diagnosed with acute bronchitis were treated with antibiotics. Several other studies have demonstrated even higher prescribing rates, with 85% in a recent Australian study and 71% in a study from the United States [24,25].

As many as 83.1% of patients diagnosed with pneumonia and 71.4% of the patients diagnosed with acute bronchitis had a CRP test performed. In previous studies, the use of CRP tests has been shown to improve the diagnosis of pneumonia [22,26]. However, CRP testing has a low validity for diagnosing pneumonia compared to a chest X-ray, and there is no agreement about where to set the cut-off point [27]. Previous studies have demonstrated that a combination of symptoms, signs, and CRP have high diagnostic value in detecting and mainly ruling out pneumonia [7]. Contrary, two reviews conclude that CRP testing has no clear diagnostic value in primary care [27,28]. Nevertheless, Falk et al. [28] state that when a doctor is in doubt about the presence of pneumonia, a CRP test can be helpful in ruling out disease. However, one can speculate if the CRP test is used too extensively in Danish general practice, as even a very low cut-off (>11 mg/L) was associated with being diagnosed with pneumonia. A large number of CRP tests performed in this present study perhaps represent a strategy to curb with the diagnostic uncertainty [29].

### Implications for clinical practice and future research

The emerging threat of antimicrobial resistance is real. Consequently, it is crucial to reduce the diagnostic misclassification of patients with LRTIs to minimise the use of antibiotics as much as possible. This study demonstrated a high use of CRP tests, and moreover, an elevated CRP level was strongly associated with being diagnosed with pneumonia. However, it can be questioned whether the use of CRP tests eliminates the diagnostic uncertainty. Future research should focus on testing other diagnostic tools, or optimising already existing ones, for improving the diagnosis and treatment of patients with LRTIs in general practice.

## Conclusion

Danish healthcare professionals are highly influenced by symptoms, signs and CRP tests when diagnosing patients with acute LRTIs in general practice.

## Supplementary Material

Regsitration Chart

## References

[1] WHO Health Organisation (WHO) [Internet]; 2014 [cited 2019 March 19]. Available from: http://www.who.int/drugresistance/documents/surveillancereport/en/

[2] Hughes D. Selection and evolution of resistance to antimicrobial drugs. IUBMB Life. 2014;66:521–529. 24933583 10.1002/iub.1278

[3] Costelloe C, Metcalfe C, Lovering A, et al. Effect of antibiotic prescribing in primary care on antimicrobial resistance in individual patients: systematic review and meta-analysis. Br Med J. 2010;340:c2096. 20483949 10.1136/bmj.c2096

[4] Aabenhus R, Siersma V, Hansen MP, et al. Antibiotic prescribing in Danish general practice 2004–13. J Antimicrob Chemother. 2016;71(8):2286–2294. 27107098 10.1093/jac/dkw117

[5] The Danish College of General Practitioners: Luftvejsinfektioner – diagnose og behandling. Klinisk vejledning for almen praksis [Respiratory tract infections – diagnosis and treatment. Guidelines for general practice]; 2014 [cited Sep 26]. Available from: https://vejledninger.dsam.dk/luftvejsinfektioner/.

[6] Aabenhus R, Hansen MP, Saust LT, et al. Characterisation of antibiotic prescriptions for acute respiratory tract infections in Danish general practice: a retrospective registry based cohort study. NPJ Prim Care Respir Med. 2017;27(1):37. 28526836 10.1038/s41533-017-0037-7 PMC5438385

[7] Hopstaken RM, Muris JWM, Knottnerus JA, et al. Contributions of symptoms, signs, erythrocyte sedimentation rate, and C-reactive protein to a diagnosis of pneumonia in acute lower respiratory tract infection. Br J Gen Pract. 2003;53(490):358–364. 12830562 PMC1314594

[8] Hansen JG. Management of acute rhinosinusitis in Danish general practice: a survey. Clin Epidemiol. 2011;3:213–216. 21857788 10.2147/CLEP.S23125 PMC3157491

[9] Aabenhus R, Jensen J, Jørgensen K, et al. Biomarkers as point-of-care tests to guide prescription of antibiotics in patients with acute respiratory infections in primary care. Cochrane Database Syst Rev. 2014;11:CD010130. 10.1002/14651858.CD010130.pub2 25374293

[10] Minnaard MC, van de Pol AC, Hopstaken RM, et al. C-reactive protein point-of-care testing and associated antibiotic prescribing. Fam Pract. 2016;33(4):408–413. 27230745 10.1093/fampra/cmw039

[11] Christensen SF, Jorgensen LC, Cordoba G, et al. Marked differences in GPs' diagnosis of pneumonia between Denmark and Spain: a cross-sectional study. Prim Care Respir J. 2013;22(4):454–458. 24248329 10.4104/pcrj.2013.00093 PMC6442862

[12] Munck AP, Hansen DG, Lindman A, et al. A Nordic collaboration on medical audit. The APO method for quality development and continuous medical education (CME) in primary health care. Scand J Prim Health Care. 1998;16(1):2–6. 9612871 10.1080/028134398750003313

[13] IBM Corp. Released 2017. IBM SPSS Statistics for Windows, Version 25.0. Armonk, NY: IBM Corp.

[14] Praktiserende Laegers Organisation. Faktaark [Organisation of General Practitioners in Denmark. Factsheet]; 2017 [cited Sep 26]. Available from: www.laeger.dk/sites/default/files/plo_faktaark_2017_oktober_2.pdf

[15] Sørensen LW. Sygeplejersker i beskaeftigelse og deres alder 2009–2017 [Nurses in job and age distribution 2009–2017]; 2018 [cited Sep 26]. Available from: https://dsr.dk/sites/default/files/50/notat_sygeplejersker_i_beskaeftigelse_og_deres_alder_2009-2017.pdf

[16] Paulsen MS, Andersen M, Munck AP, et al. Socio-economic status influences blood pressure control despite equal access to care. Fam Pract. 2012;29(5):503–510. 22234552 10.1093/fampra/cmr130

[17] Christensen HK, Kristensen T, Andersen MK, et al. Frailty characteristics and preventive home visits: an audit on elderly patients in Danish general practice. Fam Pract. 2017;34(1):57–62. 28122924 10.1093/fampra/cmw110

[18] Stuhr JK, Lykkegaard J, Kristensen JK, et al. Danish GPs' and practice nurses' management of acute sore throat and adherence to guidelines. Fam Pract. 2019;36(2):192–198. 29924311 10.1093/fampra/cmy059

[19] Strandberg EL, Ovhed I, Troein M, et al. Influence of self-registration on audit participants and their non-participating colleagues. A retrospective study of medical records concerning prescription patterns. Scand J Prim Health Care. 2005;23(1):42–46. 16025873 10.1080/02813430510018400

[20] Munck AP, Damsgaard J, Hansen DG, et al. The Nordic method for quality improvement in general practice. Qual Prim Care. 2003;11:73–78.

[21] Howie J. Diagnosis in general practice and its implications for quality of care. J Health Serv Res Policy. 2010;15(2):120–122. 19833858 10.1258/jhsrp.2009.009109

[22] Woodhead M, Blasi F, Ewig S, et al. Guidelines for the management of adult lower respiratory tract infections – full version. Clin Microbiol Infect. 2011;17(Suppl 6):E1–E59. 10.1111/j.1469-0691.2011.03672.x PMC7128977 21951385

[23] Smith S, Fahey T, Smucny J, et al. Antibiotics for acute bronchitis. Cochrane Database of Syst Rev. 2017;6:CD000245. 28626858 10.1002/14651858.CD000245.pub4 PMC6481481

[24] McCullough AR, Pollack AJ, Hansen MP, et al. Antibiotics for acute respiratory infections in general practice: comparison of prescribing rates with guideline recommendations. Med J Aust. 2017;207(2):65–69. 28701117 10.5694/mja16.01042

[25] Barnett ML, Linder JA. Antibiotic prescribing for adults with acute bronchitis in the United States, 1996–2010. J Am Med Assoc. 2014;311(19):2020–2022. 10.1001/jama.2013.286141 PMC4529023 24846041

[26] van Vugt SF, Broekhuizen BD, Lammens C, et al. Use of serum C reactive protein and procalcitonin concentrations in addition to symptoms and signs to predict pneumonia in patients presenting to primary care with acute cough: diagnostic study. Br Med J. 2013;346:f2450. 23633005 10.1136/bmj.f2450 PMC3639712

[27] van der Meer V, Neven AK, van den Broek PJ, et al. Diagnostic value of C reactive protein in infections of the lower respiratory tract: systematic review. Br Med J. 2005;331(7507):26. 15979984 10.1136/bmj.38483.478183.EB PMC558535

[28] Falk G, Fahey T. C-reactive protein and community-acquired pneumonia in ambulatory care: systematic review of diagnostic accuracy studies. Fam Pract. 2008;26(1):10–21. 19074757 10.1093/fampra/cmn095

[29] Alam R, Cheraghi-Sohi S, Panagioti M, et al. Managing diagnostic uncertainty in primary care: a systematic critical review. BMC Fam Pract. 2017;18(1):79. 28784088 10.1186/s12875-017-0650-0 PMC5545872

