# Supplementary material for: Acute lower respiratory tract infections: Symptoms, findings and management in Danish general practice
Source: Eur J Gen Pract. 2019 Oct 25;26(1):14–20. doi: 10.1080/13814788.2019.1674279 (PMC7034054; doi:10.1080/13814788.2019.1674279)
Supplement: Regsitration Chart [file IGEN_A_1674279_SM9454.pdf]

# Acute respiratory tract infections in general practice in 2017 and 2018

Registration date :

| | Age | Sex | | Duration | Symptoms | | | | | | | | | | Findings | | | | | | Examinations/ investigations | | | | | | | Your diagnosis | | | | | | | | Antibiotics | | | | | Other | | | | |
|---|---|---|---|---|---|---|---|---|---|---|---|---|---|---|---|---|---|---|---|---|---|---|---|---|---|---|---|---|---|---|---|---|---|---|---|---|---|---|---|---|---|---|---|---|---|
| | | | | | Min 1x | | | | | | | | | | Min 1x | | | | | | Min 1x | | | | | | | Only 1x | | | | | | | | Min 1x | | | | | Min 1x | | | | |
| | | | | Number of symptom days | Exacerbation of symptoms after temporary improvement | Fever (temp. > 38,5) | Cough | Purulent rhinorrhea | Purulent ear discharge | Ear/facial pain | Painful swallowing | Dyspnoea/polypnoea | Increased purulent sputum | None of the above | Swollen angular lymph nodes | Tonsillar exudates | Abnormal pulmonary auscultation/chest retractions | Unwell patient | Weakened or multimorbid patient | None of the above | Strep A positive | Strep A negative | CRP (mg/L), indicate value | Abnormal tympanometry | Normal tympanometry | Chest X-ray | None of the above | Common cold | Acute otitis media | Acute rhinosinusitis | Acute pharyngotonsillitis | Acute bronchitis | Acute exacerbation of COPD | Pneumonia | Other respiratory tract infection | Penicillin V | Amoxicillin +/÷ Clavulanic acid | Macrolide | Other antibiotic | No antibiotic | Penicillin allergy | Patient requesting a test | Patient requesting antibiotics | Referral to other specialist/hospital | None of the above |
| | | M | K | 1 | 2 | 3 | 4 | 5 | 6 | 7 | 8 | 9 | 10 | 11 | 12 | 13 | 14 | 15 | 16 | 17 | 18 | 19 | 20 | 21 | 22 | 23 | 24 | 25 | 26 | 27 | 28 | 29 | 30 | 31 | 32 | 33 | 34 | 35 | 36 | 37 | 38 | 39 | 40 | 41 | 42 |
| 1 | | | | | | | | | | | | | | | | | | | | | | | | | | | | | | | | | | | | | | | | | | | | | |
| 2 | | | | | | | | | | | | | | | | | | | | | | | | | | | | | | | | | | | | | | | | | | | | | |
| 3 | | | | | | | | | | | | | | | | | | | | | | | | | | | | | | | | | | | | | | | | | | | | | |
| 4 | | | | | | | | | | | | | | | | | | | | | | | | | | | | | | | | | | | | | | | | | | | | | |
| 5 | | | | | | | | | | | | | | | | | | | | | | | | | | | | | | | | | | | | | | | | | | | | | |
| 6 | | | | | | | | | | | | | | | | | | | | | | | | | | | | | | | | | | | | | | | | | | | | | |
| 7 | | | | | | | | | | | | | | | | | | | | | | | | | | | | | | | | | | | | | | | | | | | | | |
| 8 | | | | | | | | | | | | | | | | | | | | | | | | | | | | | | | | | | | | | | | | | | | | | |
| 9 | | | | | | | | | | | | | | | | | | | | | | | | | | | | | | | | | | | | | | | | | | | | | |
| 10 | | | | | | | | | | | | | | | | | | | | | | | | | | | | | | | | | | | | | | | | | | | | | |
| 11 | | | | | | | | | | | | | | | | | | | | | | | | | | | | | | | | | | | | | | | | | | | | | |
| 12 | | | | | | | | | | | | | | | | | | | | | | | | | | | | | | | | | | | | | | | | | | | | | |

®Copyright: Audit Projekt Odense, J.B. Winsløws Vej 9A, 1. 5000 Odense C
